# Differences in prevalence and risk factors of diabetic retinopathy among rural and urban residents with diabetes in South China: a cross-sectional study

**DOI:** 10.1136/bmjopen-2024-092526

**Published:** 2025-03-21

**Authors:** Baixiang Xiao, Fang Duan, Xuejun Gu, Jiahao Zuo, Ving Fai Chan, Gianni Virgili, Xiaojun Zhou, Carlos Price-Sanchez, Ling Jin, Yuanping Liu, Yanfang Wang, Yichun Zhong, Qinghua Liao, Haoxiang Fu, Yun He, Dongfeng Li, Ping Xu, Jia Li, Nathan Congdon

**Affiliations:** 1 Affiliated Eye Hospital, Jiangxi Medical College, Nanchang University, Nanchang City, China; 2 Center for Public Health, Queen’s University Belfast, Belfast, UK; 3 Sun Yat-Sen University Zhongshan Ophthalmic Center, Guangzhou, China; 4 School of Public Health, Nanchang University, Nanchang City, China; 5 Zhenjiang District People’s Hospital, Shaoguan City, China; 6 School of Public Health, Sun Yat-Sen University, Guangzhou, Guangdong, China; 7 Sichuan Provincial People’s Hospital, Chengdu, Sichuan, China; 8 ORBIS International, New York City, New York, USA

**Keywords:** Prevalence, Diabetic retinopathy, China, Mass Screening

## Abstract

**Abstract:**

**Objective:**

To determine the prevalence of diabetic retinopathy (DR) and associated risk factors among rural and urban people diagnosed with type 1 or type 2 diabetes mellitus (PwDM) in southern China.

**Design:**

This is a cross-sectional study.

**Setting:**

The study was conducted at primary health centres from 2 September to the end of December 2019.

**Participants:**

All the 3646 PwDM being registered in Qujiang District, aged ≥18 years were informed, of whom 2677 attended the screening and were recruited.

**Primary and secondary outcome measures:**

The presence of DR was determined by trained graders using criteria of the UK National Health Service Diabetic Eye Screening Programme. Multiple logistic regression analyses were used to assess potential risk factors for the presence of DR.

**Results:**

The mean age of the participants (73.4% of the 3646 invited people) was 63.4 years (SD 10.1 years), 1503 (56.2%) were female, 1749 (65.3%) were rural residents, 1654 (71.0%) participants had glycated haemoglobin (HbA1c)≥6.5%, 1773 (66.3%) had high blood pressure and the median duration of diabetes mellitus (DM) was <5 years. Although half (52.0%) of these participants had brief (<5 years) exposure to DM, 831 (31.3%, 95% CI: 29.3 to 32.8%) had DR, among whom 412 (49.6%) had sight-threatening DR. Men (33.1%) had a significantly higher prevalence of any DR than women (29.4%, p<0.05). There were no significant differences in DR prevalence at any level between rural and urban residents. In multivariate regression models, risk factors for any DR were HbA1c>6.5% (OR=1.58, p<0.01), using insulin and antihyperglycaemic medications (OR=1.76, p<0.01), longer duration of DM and higher systolic blood pressure (OR=1.01 for each mm Hg, p<0.01).

**Conclusions:**

The high prevalence of DR, hyperglycaemia and high blood pressure highlight the need for better management of non-communicable diseases in China.

**STRENGTHS AND LIMITATIONS OF THIS STUDY**

- This study was conducted in close cooperation with primary healthcare and enhanced the services at the level.
- We recruited a large number of participants from real-world screening practices. This made it possible to compare rural and urban people with diabetes mellitus (DM).
- Although the participation rate in the offered eye examinations was relatively high, at approximately three-quarters of the eligible persons, the presented people with DM differed from those who did not, having younger age, worse-controlled blood pressure and less urban dwellers.
- Data collected in the region of southern China and extrapolated to other regions can only be obtained with care.

## Introduction

 The global number of people with diabetes mellitus (PwDM) increased from 108 million in 1980 to 463 million in 2019, this resulting in 4.2 million deaths in the latter year.^1^,^3^ China has also observed a surge in diabetes mellitus (DM) prevalence among adults from <1% in the 1980s to 10.9% in 2010.^1 4^

Hyperglycaemia among PwDM can damage blood vessels in the retina and lead to the development of diabetic retinopathy (DR). At the early or even late stages of the disease, the damage is often asymptomatic, not affecting the central vision. This poses a significant danger to PwDM, as untreated DR can lead to vision impairment (VI) and eventual blindness (BL). However, early detection of DR via annual retinal examinations coupled with interventions such as laser retinal surgery, intraocular injection of antivascular endothelial growth factor and/or vitrectomy surgery can reduce VI by >90%.^5 6^

Among Chinese PwDM, prevalence of DR has been reported as 18.0% among urban dwellers in Eastern China during a community screening in 2015,^3^ 18.5% from pooled prevalence of a review,^7^ 27.9% from a multihospital-based study in 2015^8^ and 34% (95% CI: 33.3 to 34.9) from a six-province study in the same year.^9^ Previous studies have established risk factors for DR, including longer duration of DM, higher levels of glycated haemoglobin (HbA1c %) and serum lipids, high blood pressure, living in rural areas and use of insulin.^7 8^

Studies have reported that a large proportion of PwDM in China are undiagnosed.^3 10^ China’s National Primary Healthcare System recommends that PwDM register with township health units (THUs) in rural areas or community health centres (CHCs) in urban areas for regular monitoring of their glucose levels and potential complications. However, most THUs/CHCs do not currently offer retinal examinations due to a national lack of trained technical staff.

In 2017, the ‘Integrated Diabetic Rural Eye Care’ programme, funded by the World Diabetes Foundation (WDF) and Orbis International, was launched in Guangdong Province, southern China. The aim was to establish a model of diabetic retinopathy screening (DRS) at the primary level in the programme areas. Qujiang is a county-level district with a population of 305 500 and was included in the programme. The district has 10 towns and commenced DRS in September 2019.

Most studies on the prevalence of DR have been completed in China’s northern and eastern areas.^7 11^ One study undertaken in Guangdong Province in 2014, including only 562 participants, showed that the prevalence of DR among previously and newly diagnosed PwDM older than 50 years were 23.9% and 8.19%, respectively.^10^ While population-based data are necessary to fully understand the nature of DM in rural China, including the percentage of persons diagnosed and under care, large studies of persons already receiving treatment for DM are also important to understand the current quality of care for PwDM. Few large studies such as those in Beijing^12^,^14^ and Suzhou^3^ have been done of this type in China, fewer still in rural medical facilities and none assessing prevalence of DR using state-of-the-art image assessment by trained graders. We conducted this study on the prevalence and risk factors of DR among both rural and urban dwellers PwDM (including type 1 diabetes mellitus (T1DM) and type 2 diabetes mellitus (T2DM)) in order to inform policymakers and improve existing public health services.

## Methods

 PwDM found with DR at the preproliferative stage or beyond, any maculopathy, operable cataract or other diagnosed or suspected eye diseases were referred to Shaoguan Hospital for further examination and treatment as needed. The study fulfilled the tenets of the Declaration of Helsinki.

### Patient and public involvement

PwDM in Qujiang receives quarterly physical examination by the primary health staff, and this reported DRS was part of the regular health check. Screening was announced through posters in all villages. All PwDM registered in Qujiang (n=3646) were informed again by trained village doctors of a scheduled DRS. The questionnaires were pretested on 30 PwDM and revised based on their responses. Primary health staff’s comments on the questionnaires were taken for revision. Patients were not directly involved in the study design, data analysis and reporting.

Trained study team members at the screening sites included an ophthalmologist, two nurses, one technician and three interviewers. Participant information, including age, gender, body mass index (BMI) and history of smoking and alcohol consumption, duration of DM, had all been previously recorded and annually updated at health centres by the local general practitioners following national guidelines for the management of chronic diseases. These data were retrieved from the record system when participants arrived at the screening site. Screening began with assessment of each participant’s weight, height and blood pressure. A health questionnaire was administered, including items on official place of residence (rural vs urban), marital status, type of medical insurance, level of education, management of glucose, knowledge, attitudes and practices concerning DM, their own health and economic situation. Venous blood was drawn from each consenting participant in order to assess HbA1c and kidney function.

Hypertension was defined as presenting with systolic pressure≥140 mm Hg, diastolic pressure≥90 mm Hg or having been previously diagnosed. Normal reference value for blood urea nitrogen (BUN) is 2.9–8.2 mmol/L and creatinine (Cr) is 62–115 umol/L.

Finally, an ophthalmic examination was conducted as follows: a trained nurse tested presenting vision acuity (PVA) using an illuminated Snellen chart at 6 m. An ophthalmologist examined the anterior segment under a slit lamp and tested intra-ocular pressure (IOP) using a hand-held tonometer (Tonopen, Reichert, USA). For participants with normal IOP and without narrow angles, assessed by using the Van Herrick method,^15^ the ophthalmic nurse dilated pupils bilaterally with one to two drops of tropicamide (0.25%) 2–5 min before capturing images. Participants thought to be at risk of side effects from pharmacologic dilation, based on assessment of the anterior chamber angle, IOP>21 mm Hg (suggested by the local ophthalmologists) or a history of symptoms suggestive of acute angle closure attack, were taken to a dark room for 3–5 min to undergo scotopic pupillary dilation instead. Two fundus images were captured from each dilated or undilated eye by a trained technician. One image was centred at the optic nerve head and the other at the macula. Fundus images were captured directly without dilation for those with IOP≥21 mm Hg or who had been previously diagnosed with glaucoma, and those without reaction on scotopic pupillary dilation. If the technician was not satisfied with the first round of images taken, the process was repeated until the images were clear, unless the ophthalmologist at the screening site indicated the blurred image was due to media opacity, in which case further image capture in the eye was not attempted. All images were taken in a darkened room with a digital non-mydriatic fundus camera (Fundus Vue, Crystal Vue, Taipei, Taiwan) with a 45^0^ field of view.

Images were uploaded to an online grading system (https://grader.com) and independently assessed by two certified graders in Zhongshan Ophthalmic Centre, Sun Yat-sen University, Guangzhou City, the top eye institute in the country. These graders were trained and qualified by the ophthalmologists and had been on the role for over 5 years. Graders discussed the results and resolved any disagreements. The English National Screening Programme Standard^7^ was followed to grade the quality of images (ungradable vs gradable) and the presence and severity of DR.

The severity of each participant’s DR was determined based on the grade of their worse affected eye. If gradable images were available for only one eye, the participant’s grade depended on that eye alone. Sight-threatening DR (STDR) was defined as the presence of preproliferative DR (R2), proliferative DR (R3) and/or maculopathy (M1). If images were ungradable, the photographer at the screening site would record reasons for this in consultation with the ophthalmologist. Ungradable eyes were included in the denominator when the prevalence of DR was computed in the SPSS model in this article.

Participants with visual impairment (VI) could be classified as blind (PVA<3/60 in the better-seeing eye), severely visually impaired (SVI, PVA≥3/60, but <6/60) or moderately visually impaired (MVI) (PVA≥6/60, but <6/18). The cause(s) of VI and blindness were also determined by the ophthalmologist on site on the eye examination (DR or other ocular conditions) with torch, portable slit lamp, tono-pen and fundus camera. Should the eye have more than one comorbidity, the most easily treated condition was recorded as the cause of VI.^16^ This sequential principle is predicated on difficulty levels ranging from elementary to complex, from anterior to posterior segment, and is adapted from the principal cause selection of VI or blindness in the methodology of the Rapid Assessment of Avoidable Blindness.^16^ The following rank of the principal causes was followed: (1) refractive errors or aphakia, uncorrected; (2) untreated cataract; (3) preventable corneal opacities; (5) glaucoma and (6) other posterior segment diseases (online supplemental table 1).

All analyses were performed using a commercially available statistical software package (SPSS for Windows, V.26.0; SPSS, Chicago, IL). Prevalence of any DR, STDR, VI and blindness was calculated for all screening participants. Data were analysed using person as the unit, with the exception of burden of VI/BL, which was calculated by person and by eye. Continuous variables were presented as means (SD) or medians (IQR). Categorical variables were presented as frequencies and proportions. Duration of diagnosed diabetes and hypertension was stratified into intervals of 5 years. The Shapiro–Wilk normality test and histograms were used to evaluate the normality of continuous data. Univariate logistic regression was performed separately for potential predictors of any DR and STDR, and variables with a p-value<0.2 were included in multi-variable logistic regression models.

## Results

**Figure 1:**
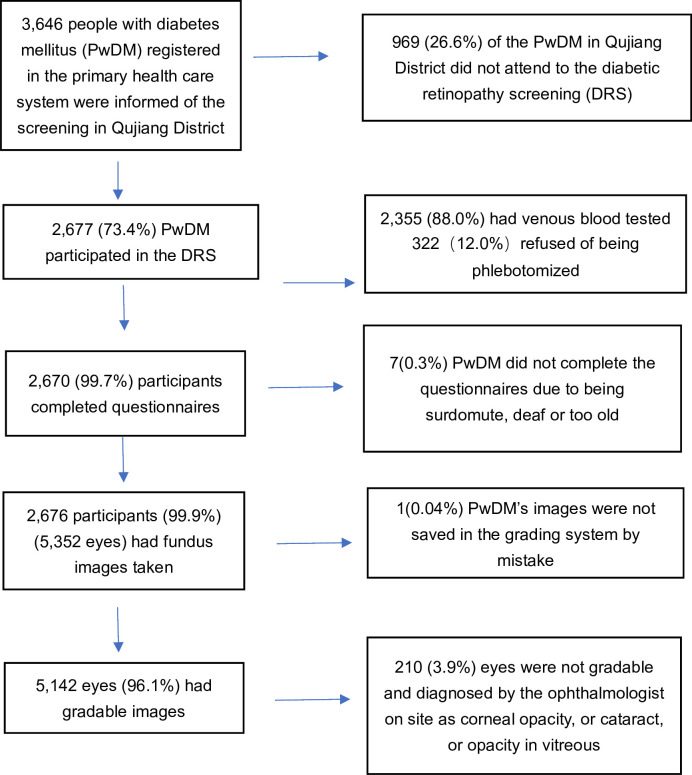
Flowchart of participants included and excluded for analysis of diabetic retinopathy in Qujiang District.

Of the 3646 PwDM contacted, 2677 (73.4%) (1503 (56.1%) female; mean age 63.4 years (SD 10.1, range 28–97 years)) participated in the screening (figure 1). Among participants, 346 (12.9%) refused to have their venous blood collected onsite, and fundus images for one person (0.04%) were not saved (figure 1). Most participants (n=2643, 98.7%) reported having type 2 DM, with 14 (0.5%) having type 1 and 20 (0.7%) uncertain. Some half (1392, 52.0%) of participants had been diagnosed with DM<5 years ago, and for 229 (8.6%), the time since diagnosis was ≥15 years. Among participants attending the DRS, 473 (17.7%) had a history of smoking, 326 (12.2%) drank alcohol regularly, 333 (12.4%) had a family history of DM and 1654 (71.0%) had HbA1c≥6.5%.

The mean age of PwDM who did not present for screening (65.2 (10.6) years) was significantly greater than for those who did (63.4 (10.1) years, p<0.01), and their mean systolic (130.6 (12.0) vs 137.0 (18.8), p<0.01) and diastolic (79.2 (7.3) vs 82.1 (11.0), p<0.01) blood pressures were significantly lower. There were no significant differences in mean BMI, gender, positive history of smoking or reported alcohol consumption between those who did and did not attend screening (table 1).

**Table 1:** Characteristics of 3646 people with diabetes mellitus who did and did not present for the screening in Qujiang District

	Total(n=3646)	Presented for screening(n=2677, 73.4%)	Did not present for screening(n=969, 26.6%)	P value
Age (years)	63.9 (10.3)	63.4 (10.1)	65.2 (10.6)	<0.01
Female sex	2037 (55.9%)	1503 (56.2%)	534 (55.1%)	0.59
Positive history of smoking	662 (18.2%)	473 (17.7%)	189 (19.5%)	0.34
Self-reported regular use of alcohol	457 (12.5%)	326 (12.2%)	131 (13.5%)	0.37
Systolic blood pressure (mm Hg)	135.3 (17.5)	137.0 (18.8)	130.6 (12.0)	<0.01
Diastolic blood pressure (mm Hg)	81.4 (10.2)	82.1 (11.0)	79.2 (7.3)	<0.01
Duration of diabetes (mean year)	6.8 (5.8)	6.3 (5.6)	7.59 (6.1)	<0.05
Body mass index (kg/m^2^)	24.4 (4.9)	24.5 (5.4)	24.3 (3.2)	0.28
Living places rural	2333 (64.1%)	1749 (65.3%)	584 (60.3%)	<0.05
Urban	1313 (35.9%)	928 (34.7%)	385 (39.7%)	

The proportions of people with MVI, SVI and blindness were 9.7% (n=260), 0.7% (n=18) and 1.0% (n=27), respectively (table 2). Among all participants, there were 382 (14.3%) persons (598 eyes, 11.2%) with at least one eye having VI, 53 (2.0%) people (71 eyes, 1.3%) with SVI and 182 (6.8%) people (208 eyes, 3.9%) with at least one eye blind.

**Table 2:** Characteristics of participants in the diabetic retinopathy screening in Qujiang, stratified by official urban versus rural residence

Characteristic	Total(n=2677)	Ruraln=1749 (65.3%)	Urbann=928 (34.7%)	P value
Mean age (SD)	63.4 (10.1)	62.4 (10.2)	65.3 (9.6)	<0.01
Age (years) (categorised)				<0.01
<50	226 (8.4%)	174 (9.9%)	52 (5.6%)	
50–59	704 (26.3%)	512 (29.3%)	192 (20.7%)	
60–69	992 (37.1%)	636 (36.4%)	356 (38.4%)	
≥70	755 (28.2%)	427 (24.4%)	328 (35.3%)	
Female sex	1503 (56.1%)	985 (56.3%)	518 (55.8%)	0.80
Self-reported history of smoking	473 (17.7%)	346 (73.1%)	127 (26.9%)	<0.01
Self-reported regular use of alcohol	326 (12.2%)	234 (13.4%)	92 (9.9%)	<0.01
Family history of DM	333 (12.4%)	209 (11.9%)	124 (13.4%)	0.29
High school and above education	322 (12.0%)	142 (8.1%)	180 (19.4%)	<0.01
Mean HbA1c (%) (SD)	7.5 (1.6)	7.4 (1.6)	7.5 (1.5)	0.10
HbA1c (%)≥6.5*	1654 (71.0%)	1061 (69.0%)	593 (74.8%)	<0.01
Mean blood urea nitrogen (BUN) (SD) (mmol/L)	6.1 (2.2)	6.1 (2.3)	6.1 (2.1)	0.64
Mean creatinine (Cr) (SD) (μmol/L)	97.6 (44.6)	97.9 (41.2)	97.1 (50.5)	0.70
Mean body mass index (BMI) (kg/m^2^) (SD)	24.5 (5.4)	24.21 (3.5)	24.36 (3.4)	0.30
Visual acuity (better-seeing eye)				
Moderate visual impairment	216 (8.1%)	146 (8.3%)	70 (7.5%)	0.47
Severe visual impairment	18 (0.7%)	10 (0.6%)	8 (0.9%)	0.38
Blindness	27 (1.0%)	21 (1.1%)	6 (0.6%)	0.21
Duration of DM				<0.01
<5 years	1392 (52.0%)	983 (56.2%)	409 (44.1%)	<0.01
≥5 years, but less than 10 years	716 (26.7%)	469 (26.8%)	247 (26.6%)	0.91
≥10 years, but less than 15 years	340 (12.7%)	199 (11.4%)	141 (15.2%)	<0.01
≥15 years	229 (8.6%)	98 (5.6%)	131 (14.1%)	<0.01
Mean systolic blood pressure (mm Hg)	137.0 (18.8)	137.7 (18.9)	135.7 (18.4)	0.01
Mean diastolic blood pressure (mm Hg)	82.1 (11.0)	82.7 (11.1)	81.0 (10.7)	<0.01
Hypertension present	1773 (66.2%)	1144 (65.4%)	629 (67.8%)	0.22
Retinal examination in the previous year	329 (12.3%)	205 (11.7%)	124 (13.4%)	0.22
Use of insulin and/or antihyperglycaemic medications	2147 (80.2%)	1385 (79.2%)	762 (82.1%)	0.07

Moderate visual impairment (MVI) presenting visual acuity (PVA)>/≥6/60, but<6/18.

Severe visual impairment (SVI) PVA>/≥3/60,<6/60.

Blindness PVA<3/60.

* HbA1C (%). This value was missing for 346 (12.9%) of participants.

BPblood pressureDMdiabetes mellitusHbA1Cglycated haemoglobin

Only 329 (12.3%) participants had received a retinal examination in the previous year. Over half (1773, 66.2%) of these PwDM had hypertension. Most participants (n=2147, 80.2%) were using insulin and/orantihyperglycaemic medications, while the rest employed some combination of Chinese traditional medicines, diet or exercise. A total of 186 (6.9%) participants reported taking no measures to manage their glucose (table 2).

Over half of those screened (n=1745, 65.3%) were rural residents. There were no significant differences between rural and urban residents with regard to gender, family history of DM, prevalence of VI and blindness, history of hypertension, BMI, HbA1c or proportion who had received a retinal examination in the previous year, nor in their use of insulin and/or antihyperglycaemic medications. However, rural dwellers were more likely to be smokers (p<0.01), to drink alcohol regularly (p<0.01) and to have been diagnosed with DM for <5 years (p<0.01). Among urban residents, a higher proportion had achieved an educational level of high school or above, had HbA1c (%) equal to or over 6.5 (p<0.01) and had been diagnosed with DM for over 15 years (table 2). The average age of urban residents (65.3 (SD 9.6) years) was significantly older than rural ones (62.4 (SD 10.2) (p<0.01)). Both mean systolic and diastolic blood pressures were significantly higher for rural residents (p<0.05) (table 2).

Of the 2676 people who had fundus images taken, 20 (0.8%) had both eyes with ungradable pictures taken. Nine of these 20 people had dense cataracts, 1 had corneal opacity in his right eye and left eyeball absent, 1 had an undilated pupil too small and the other nine had opacity in the vitreous, including asteroid hyalosis (3) or vitreous haemorrhage or fibrosis (6). Of the 5352 eyes, 168 (6.3%) had ungradable images.

A total of 831 (31.1%, 95% CI: 29.3 to 32.8) participants had any DR. The prevalence of M1 and STDR were 13.2% (95% CI: 11.9 to 14.4) and 15.4% (95%CI: 14.0 to 16.8), respectively (table 3). The prevalence of any DR, including STDR, was similar between rural and urban residents. The prevalence of both STDR (men: 17.5% vs women: 13.7%, <0.01) and any DR (men: 33.1% vs women: 29.4%, p<0.05) was significantly higher in men. There was no significant difference for any DR among the different BMI groups, while participants with a BMI over 30 had a significantly higher prevalence of maculopathy. Longer duration of DM, having higher BUN and Cr were significantly associated with greater severity and higher prevalence of all levels of DR (p<0.01).

**Table 3:** Prevalence of diabetic retinopathy in Qujiang among rural and urban residents and by age, sex and clinical status

DR grade	Any DR	R3	M1	STDR
	n (%, 95% CI)	n (%, 95% CI)	n (%, 95% CI)	n (%, 95% CI)
Total (n, %)	831 (31.1%, 29.3 to 32.8)	54 (2.0%, 1.5 to 2.6)	352 (13.2%, 11.9 to 14.4)	412 (15.4%, 14.0 to 16.8)
Residence				
Rural (n=1748, 650.3%)	535 (30.6%, 28.4 to 32.8)	36 (2.1%, 1.4 to 2.7)	231 (13.2%, 11.6 to 14.8)	271 (15.5%, 13.8 to 17.2)
Urban (n=928, 340.7%)	296 (31.9%, 28.9 to 34.9)	18 (1.9%, 1.1 to 2.8)	121 (13.0%, 10.9 to 15.2)	141 (15.2%, 12.9 to 17.5)
P value	0.49	0.83	0.89	0.83
Age (years)				
<50 (n=218, 80.1%)	73 (32.3%, 26.2 to 38.4)	6 (2.7%, 0.5 to 4.8)	32 (14.2%, 9.6 to 18.7)	40 (17.7%, 12.7 to 22.7)
50–59 (n=779, 290.1%)	258 (36.6%, 33.1 to 40.2)	18 (2.6%, 1.4 to 3.7)	123 (17.5%, 14.7 to 20.3)	140 (19.9%, 16.9 to 22.8)
60–69 (n=899, 330.6%)	310 (31.3%, 28.4 to 34.2)	19 (1.9%, 1.1 to 2.8)	127 (12.8%, 10.7 to 14.9)	152 (15.3%, 13.1 to 17.6)
70– (n=530,190.8%)	190 (25.2%, 22.1 to 28.3)	11 (1.5%, 0.6 to 2.3)	70 (9.3%, 7.2 to 11.3)	80 (10.6%, 8.4 to 12.8)
P value	<0.01	0.43	<0.01	<0.01
Sex				
Men (n=1174, 430.9%)	389 (33.1%, 30.4 to 35.8)	29 (2.5%, 1.6 to 3.4)	169 (14.4%, 12.4 to 16.4)	206 (17.5%, 15.4 to 19.7)
Women (n=1502, 560.1%)	442 (29.4%, 27.1 to 31.7)	25 (1.7%, 1.0 to 2.3)	183 (12.2%, 10.5 to 13.8)	206 (13.7%, 12.0 to 15.5)
P value	<0.05	0.14	0.09	<0.01
BMI (kg/m^2^)				
BMI<18.5	31 (30.7%, 21.5 to 39.8)	2 (2.0%, 0.8 to 4.7)	12 (11.9%, 5.5 to 18.3)	15 (14.9%, 7.8 to 21.9)
BMI≥18.5, but <24.9	489 (32.8%, 30.4 to 35.2)	37 (2.5%, 1.7 to 3.3)	221 (14.8%, 13.0 to 16.6)	268 (18.0%, 16.0 to 19.9)
BMI≥24.9, but <30	269 (28.7%, 25.8 to 31.6)	11 (1.2%, 0.5 to 1.9)	100 (10.7%, 8.7 to 12.7)	109 (11.6%, 9.6 to 13.7)
BMI≥30	42 (28.8%, 21.3 to 36.2)	4 (2.7%, 0.1 to 5.4)	19 (19.2%, 7.5 to 18.5)	20 (13.7%, 8.1 to 19.3)
P value	0.18	0.09	<0.05	<0.01
BUN (mmol/L) 715/2331				
<2.9	12 (32.4%, 16.6 to 48.3)	1 (2.7%, 0 to 8.2)	4 (10.8%, 0.3 to 21.3)	5 (13.5%, 2.0 to 25.1)
2.9–8.2	593 (29.4%, 27.4 to 31.4)	27 (1.3%, 0.8 to 1.8)	244 (12.1%, 11 to 13.9)	284 (14.1%, 12.6 to 15.6)
>8.2	110 (39.9%, 34.0 to 45.7)	16 (5.8%, 3.0 to 8.6)	55 (19.9%, 15.2 to 24.7)	63 (22.8%, 17.8 to 27.8)
P value	<0.01	<0.01	<0.01	<0.01
Creatinine (Cr) (μmol/L)				
<62	13 (34.2%, 18.4 to 50.0)	2 (5.3%, 0 to 12.7)	6 (15.8%, 3.6 to 27.9)	6 (15.8%, 3.6 to 27.9)
62–115	565 (29.0%, 26.9 to 31.0)	25 (1.3%, 0.8 to 1.8)	222 (11.4%, 10.0 to 12.8)	260 (13.3%, 11.8 to 14.8)
>115	137 (40.1%, 34.8 to 45.3)	17 (5.0%, 2.7 to 7.3)	75 (21.9%, 17.5 to 26.3)	86 (25.1%, 20.5 to 29.8)
P value	<0.01	<0.01	<0.01	<0.01
Duration of DM (years)				
<5	313 (22.5%, 20.3 to 24.7)	13 (0.9%, 0.4 to 1.4)	120 (8.6%, 7.1 to 10.1)	133 (9.6%, 8.0 to 11.1)
5–10	244 (34.1%, 30.6 to 37.6)	13 (1.8%, 0.8 to 2.8)	103 (14.4%, 11.7 to 17.0)	121 (16.9%, 14.1 to 19.7)
10–15	151 (44.4%, 39.1 to 49.7)	13 (3.8%, 1.8 to 5.9)	67 (19.7%, 15.5 to 24.0)	85 (25.0%, 20.4 to 29.6)
≥15	123 (53.7%, 47.2 to 60.2)	15 (6.6%, 3.3 to 9.8)	62 (27.1%, 21.3 to 32.9)	73 (31.9%, 25.8 to 38.0)
P value	<0.01	<0.01	<0.01	<0.01
Treatment				
Insulin	139 (47.9%, 42.1 to 53.7)	11 (3.8%, 1.6 to 6.0)	68 (23.4%, 18.5 to 28.4)	88 (30.3%, 25 to 35.7)
Antihyperglycaemic medications	582 (31.4%, 29.2 to 33.5)	37 (2.0%, 1.4 to 2.6)	243 (13.1%, 11.6 to 14.6)	276 (14.9%, 13.3 to 16.5)
No medication*	110 (20.8%, 17.3 to 24.2)	6 (1.1%, 0.2 to 2.0)	41 (7.7%, 5.5 to 10.0)	48 (9.1%, 6.6 to 11.5)
P value	<0.01	<0.05	<0.01	<0.01
Presence of hypertension				
Present	349 (32.8%, 30.0 to 35.7)	27 (2.5%, 1.6 to 3.5)	147 (13.8%, 11.8 to 15.9)	171 (16.1%, 13.9 to 18.3)
Absent	482 (29.9%, 27.6 to 32.1)	27 (1.7%, 1.0 to 2.3)	205 (12.7%, 11.1 to 14.3)	241 (14.9%, 13.2 to 16.7)
P value	0.11	0.12	0.40	0.44

* This includes using Chinese Traditional Medicine, diet and exercises.

BMIbody mass indexBUNblood urea nitrogenDMdiabetes mellitusDRdiabetic retinopathySTDRsight-threatening diabetic retinopathy

Having higher HbA1c (%), using insulin and/or antihyperglycaemic medications, receiving a retinal examination in the previous year, having a longer duration of DM, higher BUN level and having higher systolic blood pressure were independently associated with greater risk of any DR and of STDR (table 4) in multivariate logistic regression models. PwDM older than 70 years were 45% less likely to have any DR and 29% less likely to have STDR (p<0.01) (table 4) than those younger than 50. Each mm Hg elevation in systolic blood pressure increased the risk of having any DR and STDR by 1.01 times (p<0.01), and every 1% elevation in HbA1c increased the risk for any DR and STDR both by 1.26 times (p<0.01).

**Table 4:** Risk factors for any diabetic retinopathy and sight-threatening diabetic retinopathy (STDR) among diabetic persons in Qujiang

Factors	Any DR		STDR	
	Univariate analysis	Multivariate regression	Univariate analysis	Multivariate regression
	OR (95% CI)	OR (95% CI)	OR (95% CI)	OR (95% CI)
Sex				
Female	Reference	Reference	Reference	Reference
Male	1.19 (1.01 to 1.40)*	1.06 (0.88 to 1.29)	1.34 (1.09 to 1.65)**	1.23 (0.96 to 1.56)
Age (years)	0.98 (0.97 to 0.99)**	0.96 (0.96 to 0.97)**	0.97 (0.96 to 0.98)**	0.96 (0.94 to 0.97)**
Residence				
Urban	Reference		Reference	
Rural	0.94 (0.79 to 1.12)		1.02 (0.82 to 1.28)	
History of smoking				
Absent	Reference	Reference	Reference	Reference
Present	1.22 (0.99 to 1.51)	1.04 (0.77 to 1.41)	1.26 (0.97 to 1.63)	1.11 (0.781 to 1.590)
Regular alcohol use				
Absent	Reference	Reference	Reference	
Present	1.21 (0.94 to 1.54)	1.12 (0.81 to 1.54)	1.10 (0.81 to 1.51)	
Family history of DM				
Absent	Reference		Reference	Reference
Present	1.01 (0.79 to 1.29)		1.36 (1.02 to 1.83)*	1.14 (0.80 to 1.61)
Educational level				
Below high school	Reference	Reference	Reference	
High school or above	1.27 (0.99 to 1.62)*	1.29 (0.97 to 1.72)	1.15 (0.84 to 1.57)	
Retinal examination in the last year				
Absent	Reference	Reference	Reference	Reference
Present	1.51 (1.19 to 1.92)**	1.28 (0.95 to 1.64)	1.69 (1.27 to 2.25)**	1.39 (1.00 to 1.93)*
Treatment of diabetes				
No medication to control blood glucose	Reference	Reference	Reference	Reference
Using insulin and/or antihyperglycaemic medications	2.43 (1.92 to 3.08)**	1.80 (1.37 to 2.37)**	2.65 (1.88 to 3.74)**	1.76 (1.20 to 2.60)**
Duration of DM (years)				
<5	Reference	Reference	Reference	Reference
≥5, but <10	1.78 (1.46 to 2.17)**	1.62 (1.30 to 2.03)**	1.92 (1.48 to 2.51)**	1.72 (1.27 to 2.32)**
≥10, but <15	2.75 (2.15 to 3.53)**	2.63 (1.97 to 3.51)**	3.15 (2.33 to 4.27)**	3.07 (2.15 to 4.37)**
≥15	4.00 (2.99 to 5.33)**	4.40 (3.14 to 6.16)**	4.43 (3.18 to 6.16)**	5.24 (3.54 to 7.74)**
Blood urea nitrogen (BUN) (mmol/L)	1.09 (1.05 to 1.13)**	1.05 (0.99 to 1.11)	1.13 (1.08 to 1.18)**	1.10 (1.02 to 1.18)*
Creatinine (μmol/L)	1.01 (1.00 to 1.01)**	1.00 (1.00 to 1.01)	1.01 (1.00 to 1.01)**	1.00 (0.99 to 1.00)
BMI (kg/m^2^)	0.98 (0.96 to 1.00)	0.97 (0.95 to 1.00)	0.95 (0.92 to 0.98)**	0.93 (0.90 to 0.97)**
Systolic blood pressure (mm Hg)	1.01 (1.00 to 1.01)**	1.01 (1.00 to 1.02)**	1.01 (0.99 to 1.01)	1.01 (1.005 to 1.02)**
HbA1c (per %)	1.32 (1.24 to 1.39)**	1.26 (1.19 to 1.33)**	1.33 (1.24 to 1.42)**	1.26 (1.18 to 1.35)**

Note: * p*P<0.05, ** p**p<0.01.

Gender, rural vs urban residency, smoking, alcohol consumption, family history of DM and level of education were all unassociated with the risk of any DR or of STDR in multivariate logistic models.

BMIbody mass indexDMdiabetes mellitusDRdiabetic retinopathyHbA1Cglycated haemoglobinSTDRsight-threatening diabetic retinopathy

## Discussion

Although half of the PwDM in our study had DM for <5 years, our findings of 31.1% DR prevalence are comparable to recent studies in both Hong Kong and the UK through clinic-based screening, with prevalence of 39.0% (95% CI: 38.8 to 39.2), and 33.8% respectively, where PwDM had much longer duration of DM.^17 18^ This suggests that the actual duration of DM prior to diagnosis may have been longer, especially for rural people in our cohort in the present study, which is consistent with prior studies showing a low rate of prior diagnosis (14%) among PwDM in this setting.^10^ An alternative explanation for the relatively high prevalence of DR after a brief apparent period of exposure to DM may be inadequate glucose control and other DR risk factors: nearly three-quarters (71.0%) of participants in the current study had HbA1c>6.5% and two-thirds (66.2%) had uncontrolled hypertension. Higher rates of hyperglycaemia were detected among urban dwellers. This evidence of inadequate management of common non-communicable diseases, resulting in a comparatively high prevalence of end-organ damage such as DR, has important implications for health policymakers in China.

Other clinic-based studies in China examining DR prevalence among PwDM registered in clinics at different levels have reported rates from 18.0% (year 2015 in urban Suzhou, 95% CI: 15.5 to 20.6)^3^ to 27.9% (eight hospitals across China in 2016–2016, 95% CI: 27.2 to 28.6)^8^ to 32.8% (Dongguan City in Guangdong Province between 2011 and 2012).^19^ None of these studies, however, followed our gold standard approach of assessing photographic images using experienced graders, and some other studies with direct examination of the retina in clinic may have led to under-estimation of DR, as has been reported in China.^20^

Rural-dwellers in the current study were less educated and more likely to smoke and drink alcohol regularly than urban-dwellers. Nonetheless, we found no difference in DR prevalence by urban versus rural residence. In adjusting for age (younger in rural dwellers) and duration of diabetes (shorter in rural dwellers), there was still no significant difference detected in DR prevalence.

Our findings that elevated blood pressure and hyperglycaemia were associated with higher rates of DR are consistent with results from the large trials.^21 22^ When glucose and blood pressure were controlled, lower rates of end-organ diabetes damage such as DR were observed. The observed linear association between duration of DM and DR prevalence is also a common feature of DM, particularly in this setting, where glucose and blood pressure are at high levels, as noted above. While men were at greater risk of any DR and STDR than women in the univariate regression, this association was no longer significant when adjusting for other clinical and demographic factors.

Our finding that persons under current treatment with insulin/hypoglycaemic agents and those having had recent retinal examinations were at greater risk for DR likely reflects the fact that those with more severe disease, resulting in worse DR, were more likely to be receiving aggressive treatment and to have visual symptoms, leading to greater uptake of ocular examinations. The apparent decline in DR with older age (despite the fact that DR increased with duration of DM) likely reflects a similar phenomenon: those surviving to an older age with DM likely have a later onset and are thus at lower risk for end-organ damage such as DR.

This study provides important information on the quality of DM care in China, which has previously been lacking for rural hospitals. Other strengths include the large size, relatively high uptake of offered vision examinations, our ability to compare participants with non-participants using existing health records and our use of photographic images and experienced graders to determine the prevalence of DR. The latter has not been done before in studies of diabetes management in rural China.

Limitations must also be addressed. Though participation in the offered eye examinations was relatively high at approximately three-quarters of those eligible, persons who presented differed from those who did not, having both younger age and worse-controlled blood pressure. We also missed the undiagnosed PwDM and those diagnosed but were not registered at primary healthcare or did not come for the DRS. A population-based survey would fill the gap to determine how many people were missed by the primary health-centre-based registry. While data were collected at 10 THUs, all of these facilities were in the same region of Guangdong Province, southern China, and extrapolation to other regions can only be made with care. Two images of 45^0^ only allow a small amount of the retina to be viewed and not using optical coherence tomography (OCT) means no macula thickness could be quantified in the case of maculopathy. Maculopathy is normally over-reporting on retinal photographs, while PPDR and PDR may be under-reported without wide-field imaging. Screening results are also not diagnostic so following up with ophthalmology treatment data for those with STDR is another limitation. By examining DR rates in a mixed population of T1DM and T2DM without data showing the proportion of each, and our population is likely to be dominated by T2DM. Therefore, the results are skewed towards this population and may not be true for the T1DM population.

Despite its limitations, our finding of high DR prevalence with relatively brief exposure to DM, together with high rates of inadequate glucose and BP control, delivers an important message to healthcare policymakers in China on the need to improve the management of non-communicable diseases in this setting.

## supplementary material

10.1136/bmjopen-2024-092526 online supplemental file 1

## Data Availability

Data are available upon reasonable request.
