# Supplementary material for: Differences in prevalence and risk factors of diabetic retinopathy among rural and urban residents with diabetes in South China: a cross-sectional study
Source: BMJ Open. 2025 Mar 21;15(3):e092526. doi: 10.1136/bmjopen-2024-092526 (PMC11931899; doi:10.1136/bmjopen-2024-092526)
Supplement: online supplemental file 1 [file bmjopen-15-3-s001.docx]

| **D. Main cause of presenting VA<6/12** | | | | | | | **Principal cause in** | | |
| --- | --- | --- | --- | --- | --- | --- | --- | --- | --- |
| *(Mark only one cause for each eye)* | | | | | | |  |  |  |
|  | **Right eye** | | | **Left eye** | | | **Person** | | |
| Refractive error |  |  | (1) |  |  | (1) |  |  | (1) |
| Aphakia, uncorrected |  |  | (2) |  |  | (2) |  |  | (2) |
| Cataract, untreated |  |  | (3) |  |  | (3) |  |  | (3) |
| Cataract surgical complications |  |  | (4) |  |  | (4) |  |  | (4) |
| Trachoma corneal opacity |  |  | (5) |  |  | (5) |  |  | (5) |
| Other corneal opacity |  |  | (6) |  |  | (6) |  |  | (6) |
| Phthisis |  |  | (7) |  |  | (7) |  |  | (7) |
| Onchocerciasis |  |  | (8) |  |  | (8) |  |  | (8) |
| Glaucoma |  |  | (9) |  |  | (9) |  |  | (9) |
| Diabetic retinopathy |  |  | (10) |  |  | (10) |  |  | (10) |
| ARMD |  |  | (11) |  |  | (11) |  |  | (11) |
| Other posterior segment |  |  | (12) |  |  | (12) |  |  | (12) |
| All globe/CNS abnormalities |  |  | (13) |  |  | (13) |  |  | (13) |

**Supplemental table 1**

**Part D. of the study form for the Rapid Assessment of Blindness (RAAB)**

(Abstracted from the mannual for the RAAB, available at https://iceh.lshtm.ac.uk/raab/)
