# Supplementary material for: Differences in prevalence and risk factors of diabetic retinopathy among rural and urban residents with diabetes in South China: a cross-sectional study
Source: BMJ Open. 2025 Mar 21;15(3):e092526. doi: 10.1136/bmjopen-2024-092526 (PMC11931899; doi:10.1136/bmjopen-2024-092526)
Supplement: Supplementary file 2 [file bmjopen-2024-092526.reviewer_comments.pdf]

PEER REVIEW HISTORY

BMJ Open publishes all reviews undertaken for accepted manuscripts. Reviewers are asked to complete a checklist review form and are provided with free text boxes to elaborate on their assessment. These free text comments are reproduced below.

ARTICLE DETAILS

Title (Provisional)

Differences of prevalence and risk factors of diabetic retinopathy among rural and urban residents with T1DM or T2DM in South China – A cross-sectional study

Authors

Xiao, Baixiang; Duan, Fang; Gu, Xuejun; Zuo, Jiahao; Chan, Ving Fai; Virgili, Gianni; Zhou, Xiaojun; Price-Sanchez, Carlos; Jin, Ling; Liu, Yuanping; Wang, Yanfang; Zhong, Yichun; Liao, Qinghua; Fu, Haoxiang; He, Yun; LI, DONGFENG; Xu, Ping; Li, Jia; Congdon, Nathan

---

VERSION 1 - REVIEW

**Reviewer**	**1**
**Name**	**Flaxman, Abraham**
**Affiliation**	**University of Washington**
**Date**	**28-Sep-2024**
**COI**	**None**

This paper provides a valuable descriptive epidemiological study of diabetic retinopathy (DR) in Qujiang, China. The authors drew from a sample frame of people living with diabetes mellitus (PwDR) who were diagnosed and registered in primary health centers. They found that DR is common among PwDR, with 31% prevalence among 2,677 respondents. They also found that about half of the DR is vision threatening, and investigated disparities due to urbanicity, age, and other factors. This will provide a valuable benchmark for efforts to improve the management of non-communicable diseases.

It strikes me as novel to have a reference frame from primary health centers with which to conduct such a study. Perhaps the authors should emphasize this more, to inspire future studies that use a similar design. I do wonder if there is a risk of missing undiagnosed PwDR, or people who have been diagnosed but are not captured by the registry for some reason. Is there any way to assess how many people are missed by your primary-health-center-based registry?

Minor suggestions:

Abstract: it might help reader to signpost more clearly why the 3,646 PwDM who you informed (page 3, line 10) are not all participants (2,677 participants noted in page 3, line 17); it becomes clear in the main text, but it attracted my attention unnecessarily when I was first reading the abstract.

Page 6, line 34: your decision to select the more easily treated condition as the cause of vision impairment surprised me; I understand that you need to distinguish somehow, but if you insist on this approach perhaps you could include a table of the conditions from easiest to hardest to treat. Or you could split proportionally, so that e.g. if there are two causes, you attribute the impairment 50% to one and 50% to the other.

Page 7, line 52: I was surprised to read the patients with higher BMI had lower levels of DR. Is this consistent with other investigations? Any idea why?

Page 8: I wonder if there is a figure or "infographic" that you could include to summarize the findings and important null results that you have conveyed in text --- that would make sure busy readers take away your key message.

**Reviewer**	**2**
**Name**	**Albert, Stewart**
**Affiliation**	**Saint Louis University School of Medicine, Internal Medicine**
**Date**	**29-Sep-2024**
**COI**	**None**

The authors performed screening of rural versus urban people with diabetes and confirmed that duration of diabetes, severity of diabetes and systolic hypertension are associated with diabetic retinopathy, all of which is known.

However 25% of the eligible population did to take the exams, and possible they were also less adherent to diabetes management. Table 1 should also give details of a1c, duration of diabetes and use of insulin/hypoglycemic agents comparing rural vs urban.

What is important is the evidence of recognition of new diagnoses in rural vs urban population that was not diagnosed previously. If 11% to13 % of people had retinal screening in the previous year, how many new cases were picked up in rural vs urban? Should there be mass screenings available? How would you quantify the benefit of yearly screening? Please include in table 2 or in new table and in abstract.

Reviewer **3**

**Name**	**Thomas, RL**
**Affiliation**	**Swansea University, Diabetes Resaerch Unit, Cymru**
**Date**	**22-Oct-2024**
**COI**	**None**

The authors present an important epidemiological study on diabetic retinopathy in a province of China. The study highlights critical public health information, such as the differences between rural and urban populations, including higher rates of smoking and alcohol consumption in rural areas. However, several issues need to be addressed. For instance, significant differences in variables are reported within DR groups rather than across them, and discrepancies in the logistic regression results are described that are not fully supported by the analysis provided.

Detailed feedback on the necessary revisions is outlined below.

Abstract

What type of diabetes did the population have?

If all participants had Type 2 diabetes, please specify this in the title.

Design

"This cross-sectional study informed all..." – "Informed" is not the correct term here. Consider replacing it with "included" or "enrolled."

Results

In the methods section, you mention 3,646 PwDM (People with Diabetes Mellitus), but later state there were 2,677 participants. Please clarify the reason for this discrepancy.

"Uncontrolled high blood pressure" – The term "uncontrolled" isn't necessary. Simply use "high blood pressure."

If 71% of participants had HbA1c ≥6.5%, then does that imply that 29% had an HbA1c in the prediabetes range? This should be clarified.

When reporting logistic regression, "HbA1C" should be consistently written as "HbA1c."

"Hypoglycaemic drugs" is incorrect terminology. These should be referred to as "antihyperglycaemic medications," as their goal is to lower blood glucose, not to cause hypoglycemia.

Conclusions

"Pooorly controlled blood glucose" is stigmatizing language. Please rephrase it to "hyperglycaemia" or "high blood glucose levels." The same applies to "uncontrolled hypertension"—this should be changed to "high blood pressure."

Strengths and Limitations

"which has previously lacking" should be changed to "which has previously been lacking."

"We recruited a large amount of participants" should be "We recruited a large number of participants."

Bullet Point 3 needs rewording for clarity.

Bullet Point 4 also needs rewording for clarity.

Introduction

"4.2 million deaths in that year" – Please specify the year, as the sentence contains two dates.

Avoid using "uncontrolled" when referring to diabetes. Replace this with "high glucose levels" or "hyperglycaemia."

The second sentence in the 4th paragraph ("The prevalence of DR among diagnosed PwDM…") repeats information from the previous paragraph, so it's not necessary here.

Patient and Public Involvement Statement

This section refers to patient and public involvement (PPI) in the design, conduct, and analysis of the study, not their participation as study subjects. If patients were not involved in the conduct, design, or analysis of the study, please explicitly state this.

Page 5, Line 37

"Methods for control of glucose" should be revised to "management of glucose," "management of hyperglycaemia," or "treatment of hyperglycaemia."

Line 40

"HbA1C" is incorrect and should be written as "HbA1c."

Line 50

You mention that for eyes with IOP <21 mmHg, one image was centered at the optic nerve and the other at the macula. However, for IOP ≥21 mmHg, you haven't specified which images were captured. Please clarify this.

Page 6, Line 14

Please state where the graders were based, describe their training, and clarify whether any quality assurance processes were in place.

Line 26

Ungradeable images should not be included in the denominator. Ungradeable images must be removed, and prevalence should be calculated without them.

Line 56

You report the number of people with Type 1 and Type 2 diabetes. While the number with Type 1 diabetes may be low, due to the different incidence and progression rates in these populations, it would be beneficial to include this distinction as a variable in your tables.

Page 7, Line 18

"Hypoglycaemic drugs" should be replaced with "antihyperglycaemic agents."

Line 20

The word "control" should be changed to "manage" their glucose levels.

Line 37

"The 2,676 people who had fundus images taken…" The word "who" should be added for clarity. Additionally, the sentence should be rewritten for clarity: for example, "both eyes had ungradeable images taken."

Line 41

The sentence about unilateral eyes is confusing and should be rewritten.

Lines 44-47

There are too many numerical details. If these are all included in Table 3, it would be better to mention only the most significant values in the text, such as "any DR" and "STDR."

Table 3, p-values

If the p-value is <0.05, the exact value should be reported. You should only report "<0.01" when appropriate. Additionally, it is unusual to perform significance tests within DR groups; it's typically done across groups to show differences between, for example, "No DR," "Any DR," "NSTDR," and "STDR." Your p-values may not reflect what you've stated in Lines 51-54.

Page 7, Line 60

You mention that people over 70 are 45% less likely to have any DR and 29% less likely to have STDR compared to those younger than 50. However, in Table 4, age is treated as a continuous variable. This discrepancy needs to be addressed.

Page 8, Lines 13-15

Either move this section up to where you discuss VI results, or remove it entirely. It doesn't flow well at the end.

Discussion

Page 8, Line 28

"Control of glucose" should be revised. "Poor glucose control" also needs changing—consider "higher rates of hyperglycaemia."

Line 31

The phrase "evidence of poor management" should be replaced with less stigmatizing language.

Lines 42-43

Clarify whether the methods used led to under-reporting in your study, or if the lack of these methods in other studies led to under-reporting. Also, were the populations in other studies predominantly Type 1 or Type 2 diabetes patients? This would affect the expected prevalence rate, and other population differences should be highlighted.

Line 52

"Pooorly controlled blood glucose" should be revised to "high blood glucose" or "hyperglycaemia."

Line 55

"Diabetic" should be replaced with "diabetes" in this context.

Page 9, Lines 9-12

Saying that older people have less severe disease is misleading. Diabetes is diabetes regardless of age, but the duration lived with the disease is shorter in older populations. This suggests an interaction effect that you should account for.

Additional Limitations - 2 images of 45 degrees only allows a small amount of the retina to be viewed and not using OCT means no macula thickness could be quantified in the case of maculopathy. Maculopathy is normally over reporting on retinal photographs while PPDR and PDR may be underreported without wide field imaging. Screening results are also not diagnostic so following up with ophthalmology treatment data for those with STDR is another limitation.

Line 29

This sentence needs to be rewritten for clarity.

VERSION 1 - AUTHOR RESPONSE

Reviewer: 1
Dr. Abraham Flaxman, University of Washington
Comments to the Author:
This paper provides a valuable descriptive epidemiological study of diabetic retinopathy (DR) in Qujiang, China. The authors drew from a sample frame of people living with diabetes mellitus (PwDR) who were diagnosed and registered in primary health centers. They found that DR is common among PwDR, with 31% prevalence among 2,677 respondents. They also found that about half of the DR is vision threatening, and investigated disparities due to urbanicity, age, and other factors. This will provide a valuable benchmark for efforts to improve the management of non-communicable diseases.

Many thanks for these positive comments.

It strikes me as novel to have a reference frame from primary health centers with which to conduct

such a study. Perhaps the authors should emphasize this more, to inspire future studies that use a similar design. I do wonder if there is a risk of missing undiagnosed PwDR, or people who have been diagnosed but are not captured by the registry for some reason. Is there any way to assess how many people are missed by your primary-health-center-based registry?

These are all excellent questions. There must be some missing people with DR for those who were not diagnosed with DM and those who did not attend the screening. A population study in comparison with this study would fill the gap of assessing how many people are missed by the primary-health-center-based registry. These points are now added to the discussions.

Minor suggestions:

Abstract: it might help reader to signpost more clearly why the 3,646 PwDM who you informed (page 3, line 10) are not all participants (2,677 participants noted in page 3, line 17); it becomes clear in the main text, but it attracted my attention unnecessarily when I was first reading the abstract.

Thanks. 3646 people were added to the results.

Page 6, line 34: your decision to select the more easily treated condition as the cause of vision impairment surprised me; I understand that you need to distinguish somehow, but if you insist on this approach perhaps you could include a table of the conditions from easiest to hardest to treat. Or you could split proportionally, so that e.g. if there are two causes, you attribute the impairment 50% to one and 50% to the other.

Thanks for this opinion on this matter. Regarding a table of the conditions from easiest to hardest to treat, I have now given a reference to it.

Page 7, line 52: I was surprised to read the patients with higher BMI had lower levels of DR. Is this consistent with other investigations? Any idea why?

Thanks for pointing this out. I went through the tables and related wording. Changes have now been made.

Page 8: I wonder if there is a figure or "infographic" that you could include to summarize the findings and important null results that you have conveyed in text --- that would make sure busy readers take away your key message.

I understand a figure is more clear, while there was one sentence only for the null results. I would like to leave this. Thanks.

Reviewer: 2
Dr. Stewart Albert, Saint Louis University School of Medicine
Comments to the Author:
The authors performed screening of rural versus urban people with diabetes and confirmed that duration of diabetes, severity of diabetes and systolic hypertension are associated with diabetic retinopathy, all of which is known.

Yes. Thanks for this comment. The report is based on a real screening activities and this might be different, and it was in different areas.

However 25% of the eligible population did to take the exams, and possible they were also less adherent to diabetes management. Table 1 should also give details of a1c, duration of diabetes and use of insulin/hypoglycemic agents comparing rural vs urban.

This is a good point. We did HbA1c tests for participants who agreed to have blood withdrawal in this study, while the routine records from the chronic diseases administration system tested the

fast glucose, some people had only random glucose recorded, and some PwDM even without any records, making the comparison impossible. I have now added the duration of diabetes and the different dwelling places by rural and urban.

What is important is the evidence of recognition of new diagnoses in rural vs urban population that was not diagnosed previously. If 11% to13 % of people had retinal screening in the previous year, how many new cases were picked up in rural vs urban? Should there be mass screenings available? How would you quantify the benefit of yearly screening? Please include in table 2 or in new table and in abstract.

This is really a good point and might be for the future studies. The ones had previous retinal screening were those went to hospital, rather at the primary level. We did not collect data for the matter of new case detected and it would be our future study topic.

Reviewer: 3
Dr. RL Thomas, Swansea University
Comments to the Author:
The authors present an important epidemiological study on diabetic retinopathy in a province of China. The study highlights critical public health information, such as the differences between rural and urban populations, including higher rates of smoking and alcohol consumption in rural areas. However, several issues need to be addressed. For instance, significant differences in variables are reported within DR groups rather than across them, and discrepancies in the logistic regression results are described that are not fully supported by the analysis provided.

Detailed feedback on the necessary revisions is outlined below.
Abstract
What type of diabetes did the population have?
If all participants had Type 2 diabetes, please specify this in the title.

These were not just T2DM, T1DM is also included, if PwDM is registered in the system. This is now added to the title.

Design
"This cross-sectional study informed all…" – "Informed" is not the correct term here. Consider replacing it with "included" or "enrolled."
3646 people informed and 2677 attended/included/enrolled/recruited in the study.

Results
In the methods section, you mention 3,646 PwDM (People with Diabetes Mellitus), but later state there were 2,677 participants. Please clarify the reason for this discrepancy.

Again, please refer to figure 1 for the included and excluded people. 3646 people registered in the health system and they were informed of the screening, 2677 of these informed people attended to the screening and were recruited.

"Uncontrolled high blood pressure" – The term "uncontrolled" isn't necessary. Simply use "high blood pressure."

Changed.

If 71% of participants had HbA1c ≥6.5%, then does that imply that 29% had an HbA1c in the prediabetes range? This should be clarified.

No. All the participants were diagnosed with DM, not prediabetes. Those 29% just had good glucose control.

When reporting logistic regression, "HbA1C" should be consistently written as "HbA1c."

Corrected.

"Hypoglycaemic drugs" is incorrect terminology. These should be referred to as "antihyperglycaemic medications," as their goal is to lower blood glucose, not to cause hypoglycemia.

Many thanks for your suggestion of this good clinical terminology.

Conclusions
"Pooorly controlled blood glucose" is stigmatizing language. Please rephrase it to "hyperglycaemia" or "high blood glucose levels." The same applies to "uncontrolled hypertension"—this should be changed to "high blood pressure."

Changed. Thanks.

Strengths and Limitations

"which has previously lacking" should be changed to "which has previously been lacking." "We recruited a large amount of participants" should be "We recruited a large number of participants."
Corrected. Thanks.

Bullet Point 3 needs rewording for clarity.
Bullet Point 4 also needs rewording for clarity.

These have now been improved.

Introduction

"4.2 million deaths in that year" – Please specify the year, as the sentence contains two dates.
It is now clarified.

Avoid using "uncontrolled" when referring to diabetes. Replace this with "high glucose levels" or "hyperglycaemia." Replaced.

The second sentence in the 4th paragraph ("The prevalence of DR among diagnosed PwDM…") repeats information from the previous paragraph, so it's not necessary here.

This is now deleted.

Patient and Public Involvement Statement
This section refers to patient and public involvement (PPI) in the design, conduct, and analysis of the study, not their participation as study subjects. If patients were not involved in the conduct, design, or analysis of the study, please explicitly state this.
Patients were not involved in the design, analysis and these are now added. Thank for the point.

Page 5, Line 37
"Methods for control of glucose" should be revised to "management of glucose," "management of hyperglycaemia," or "treatment of hyperglycaemia."

Amended. Thanks.

Line 40
"HbA1C" is incorrect and should be written as "HbA1c."

Thanks. This is now corrected.

Line 50
You mention that for eyes with IOP <21 mmHg, one image was centered at the optic nerve and the other at the macula. However, for IOP ≥21 mmHg, you haven't specified which images were captured. Please clarify this. Two images were taken for dilated or un-dilated eyes. This is now added.

Page 6, Line 14
Please state where the graders were based, describe their training, and clarify whether any quality assurance processes were in place. Thanks. These are now added.

Line 26
Ungradeable images should not be included in the denominator. Ungradeable images must be removed, and prevalence should be calculated without them.

As we have ophthalmologist on the screening site and all the eyes with ungradable images were detected by the ophthalmologists. Reasons of being ungradable were categorized. Ophthalmologist can tell whether the ungradable images were due to DR. For all these reasons, we would like to keep the ungradable ones in the denominator, as they are people with DM.

Line 56
You report the number of people with Type 1 and Type 2 diabetes. While the number with Type 1 diabetes may be low, due to the different incidence and progression rates in these populations, it would be beneficial to include this distinction as a variable in your tables.

Thanks for this good point. As we have too many information in this article, there were only 14 (0.5%) of the participants with T1DM, would be difficult to compare. We may do a different study on this matter in the future.

Page 7, Line 18
"Hypoglycaemic drugs" should be replaced with "antihyperglycaemic agents."

Thanks. This is now corrected.

Line 20
The word "control" should be changed to "manage" their glucose levels.
changed.

Line 37
"The 2,676 people who had fundus images taken…" The word "who" should be added for clarity. Additionally, the sentence should be rewritten for clarity: for example, "both eyes had ungradeable images taken."
Thanks for these good points. Corrected.

Line 41
The sentence about unilateral eyes is confusing and should be rewritten.
Corrected. Thanks.

Lines 44-47
There are too many numerical details. If these are all included in Table 3, it would be better to mention only the most significant values in the text, such as "any DR" and "STDR."

Thanks. Although figures were kept still in Table 3, while the description is shortened. On page 7.

Table 3, p-values
If the p-value is <0.05, the exact value should be reported. You should only report "<0.01" when appropriate. Additionally, it is unusual to perform significance tests within DR groups; it's typically

done across groups to show differences between, for example, "No DR," "Any DR," "NSTDR," and "STDR." Your p-values may not reflect what you've stated in Lines 51-54.

We compared any DR, STDR, etc. among rural and urban dwellers, different age groups….

Page 7, Line 60
You mention that people over 70 are 45% less likely to have any DR and 29% less likely to have STDR compared to those younger than 50. However, in Table 4, age is treated as a continuous variable. This discrepancy needs to be addressed.

Thanks for your notification on such detailed comments. You are right. When we input the categorized age groups in the model, the discrepancy emerged and that was the reason we treated age as the continuous variable.

Page 8, Lines 13-15
Either move this section up to where you discuss VI results, or remove it entirely. It doesn't flow well at the end.

Amended.

Discussion
Page 8, Line 28
"Control of glucose" should be revised. "Poor glucose control" also needs changing—consider "higher rates of hyperglycaemia."

Line 31
The phrase "evidence of poor management" should be replaced with less stigmatizing language.

Thanks. This is now improved.

Lines 42-43
Clarify whether the methods used led to under-reporting in your study, or if the lack of these methods in other studies led to under-reporting. Also, were the populations in other studies predominantly Type 1 or Type 2 diabetes patients? This would affect the expected prevalence rate, and other population differences should be highlighted.

Thanks. That refer to other studies. T1DM is rare in China and none of these studies separated T1DM and T2DM.

Line 52
"Pooorly controlled blood glucose" should be revised to "high blood glucose" or "hyperglycaemia."

Thanks. Corrected.

Line 55
"Diabetic" should be replaced with "diabetes" in this context.

Thanks. Corrected.

Page 9, Lines 9-12
Saying that older people have less severe disease is misleading. Diabetes is diabetes regardless of age, but the duration lived with the disease is shorter in older populations. This suggests an interaction effect that you should account for.

Agree. Thanks. This is now revised.

Additional Limitations - 2 images of 45 degrees only allows a small amount of the retina to be

viewed and not using OCT means no macula thickness could be quantified in the case of maculopathy. Maculopathy is normally over reporting on retinal photographs while PPDR and PDR may be underreported without wide field imaging. Screening results are also not diagnostic so following up with ophthalmology treatment data for those with STDR is another limitation.

Thank you so much for this good point.

Line 29
This sentence needs to be rewritten for clarity. Thanks. Amended.

Reviewer: 1
If you have selected ‘Yes’ above, please provide details of any competing interests.: Not applicable

Reviewer: 2
If you have selected ‘Yes’ above, please provide details of any competing interests.: Not applicable

Reviewer: 3
If you have selected ‘Yes’ above, please provide details of any competing interests.: N/A

VERSION 2 - REVIEW

**Reviewer**	**1**
**Name**	**Flaxman, Abraham**
**Affiliation**	**University of Washington**
**Date**	**05-Dec-2024**
**COI**	

This revision has addressed my concerns. It is possible that some of the edits described in the response to reviewers did not make it into the resubmitted document, however (discussion of how a population-based study could determine if much is missed by this study design, reference to ordering of conditions from easiest to hardest to treat).

**Reviewer**	**3**
**Name**	**Thomas, RL**
**Affiliation**	**Swansea University, Diabetes Resaerch Unit, Cymru**
**Date**	**16-Dec-2024**
**COI**	

The authors have revised the manuscript to satisfactorily answer all of my previous comments.

Very minor addition comments:

T1DM and T2DM added to title are not needed in the title especially as abbreviations.But this needs to be included In the abstract.

Abstract results: 2,677 participants – I think this could still be made clearer that 3,646 were informed but 2,677 attended screening and were included in the study.

Conclusion hyperglycaemia is spelt wrong.

Results: can you say how many of the 2677 people had type 1 and type 2 diabetes?

Discussion – you've added in poor glucose control this is not in keeping with language matters. Should be something like high glucose levels

Limitations: by looking at retinopathy rates in a mixed population of type 1 and type 2 diabetes without saying how many had each etc your population is likely to be dominated by type 2 diabetes and so results skewed towards this population and maynot hold true for type 1 diabetes. This needs to be acknowledged.

VERSION 2 - AUTHOR RESPONSE

Reviewer: 1
Dr. Abraham Flaxman, University of Washington
Comments to the Author:
This revision has addressed my concerns. It is possible that some of the edits described in the response to reviewers did not make it into the resubmitted document, however (discussion of how a population-based study could determine if much is missed by this study design, reference to ordering of conditions from easiest to hardest to treat).

Response: Thanks for your kind comments.

Reviewer: 3
Dr. RL Thomas, Swansea University
Comments to the Author:
The authors have revised the manuscript to satisfactorily answer all of my previous comments.

Very minor addition comments:
T1DM and T2DM added to title are not needed in the title especially as abbreviations. But this needs to be included In the abstract.

Response: Thanks. These are now changed.

Abstract results: 2,677 participants – I think this could still be made clearer that 3,646 were informed but 2,677 attended screening and were included in the study.

Response: Thanks. This is now modified.

Conclusion hyperglycaemia is spelt wrong.

Response: Corrected.

Results: can you say how many of the 2677 people had type 1 and type 2 diabetes?

Response: We did not keep the records as the registration system does not separate T1DM and T2DM,

Discussion – you've added in poor glucose control this is not in keeping with language matters. Should be something like high glucose levels

Response: Corrected.

Limitations: by looking at retinopathy rates in a mixed population of type 1 and type 2 diabetes without saying how many had each etc your population is likely to be dominated by type 2 diabetes and so results skewed towards this population and maynot hold true for type 1 diabetes. This needs to be acknowledged.

Response: Thanks for this good point and it is now added.

Reviewer: 1
If you have selected 'Yes' above, please provide details of any competing interests.: Not applicable

Response: Thanks. Noted and modified.

Reviewer: 3
If you have selected 'Yes' above, please provide details of any competing interests.: N/A

Response: Thanks. Changed.

VERSION 3 - AUTHOR RESPONSE

Reviewer comment: It strikes me as novel to have a reference frame from primary health centers with which to conduct such a study. Perhaps the authors should emphasize this more, to inspire future studies that use a similar design. I do wonder if there is a risk of missing undiagnosed PwDR, or people who have been diagnosed but are not captured by the registry for some reason. Is there any way to assess how many people are missed by your primary-health-center-based registry?

Response: These are all excellent questions. There must be some missing people with DR for those who were not diagnosed with DM and those who did not attend the screening. A population study in comparison with this study would fill the gap of assessing how many people are missed by the primary-health-center-based registry. These points are now added to the discussions.

VERSION 4 - AUTHOR RESPONSE

Reviewer comment: Page 6, line 34: your decision to select the more easily treated condition as the cause of vision impairment surprised me; I understand that you need to distinguish somehow, but if you insist on this approach perhaps you could include a table

of the conditions from easiest to hardest to treat. Or you could split proportionally, so that e.g. if there are two causes, you attribute the impairment 50% to one and 50% to the other.

Response:
This sequential principle is predicated on difficulty levels ranging from elementary to complex, from anterior to posterior segment, and is adapted from the principal cause selection of visual impairment or blindness in the methodology of the Rapid Assessment of Avoidable Blindness.The following rank of the principal causes was followed: 1) refractive errors or aphakia, uncorrected; 2) untreated cataract; 3) preventable corneal opacities; 5) Glaucoma; 6) Other posterior segment diseases (Supplemental table 1). The supplemental table is now uploaded.
